# Supplementary material for: High-Throughput Quantitative Screening of Glucose-Stimulated Insulin Secretion and Insulin Content Using Automated MALDI-TOF Mass Spectrometry
Source: Cells. 2023 Mar 9;12(6):849. doi: 10.3390/cells12060849 (PMC10047017; doi:10.3390/cells12060849)
Supplement: Supplementary file 1 [file cells-12-00849-s001.zip › cells-2252194-supplementary.pdf]

Supplementary Data

Table S1. List of siRNA used for Min6 cells transfection

Target Gene	Species	Product name	Product Reference	Provider	Sequence
siRNA Control	Mouse	ON-TARGETplus Non-targeting Pool	D-001810-10-20	Dharmacon	UGGUUUACAUGUCGACUAA
					UGGUUUACAUGUUGUGUGA
					UGGUUUACAUGUUUUCUGA
					UGGUUUACAUGUUUUCCUA
siKcnj11	Mouse	ON-TARGETplus SMARTpool siRNA Kcnj11	L-042183-00-0005, 16514, J-042183-05	Dharmacon	GAGAAUGGCGUGGGUGGUA
			L-042183-00-0005, 16514, J-042183-06		CAUUAUCCCUGAGGAAUAU
			L-042183-00-0005, 16514, J-042183-07		GCUAUUCUGUGGACUACUC
			L-042183-00-0005, 16514, J-042183-08		CUACCUAGCUGACGAGAUU
siPdx1	Mouse	ON-TARGETplus SMARTpool siRNA Pdx1	L-040402-01-0005, 18609, J-040402-09	Dharmacon	CUUCUGAUGCCAAGCGAAU
			L-040402-01-0005, 18609, J-040402-10		GAGCAAGAUUGUGCGGUGA
			L-040402-01-0005, 18609, J-040402-11		AAGAAACGUAGUAGCGGGA
			L-040402-01-0005, 18609, J-040402-12		GGUACAAACUUGAGCGUUC

Table S2. List of oligonucleotides used in qPCR experiments

Official symbol	Species	Sequence	Forward / Reverse
CYPA (Cyclo)	Mouse	ATGGCACTGGCGGCAGGTCC	Forward
		TTGCCATTCCTGGACCCAAA	Reverse
Pdx1	Mouse	ATTGTGCGGTGACCTCGGGC	Forward
		GATGCTGGAGGGCTGTGGCG	Reverse

Delannoy et al. Figure S1

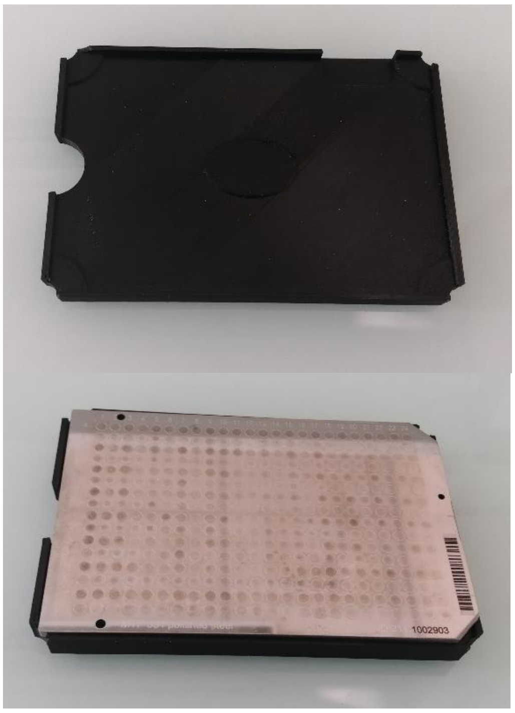

Figure S1: 3D Printing adapter to allow the use of the MALDI target by Biomek NXP liquid handler

Delannoy et al. Figure S2

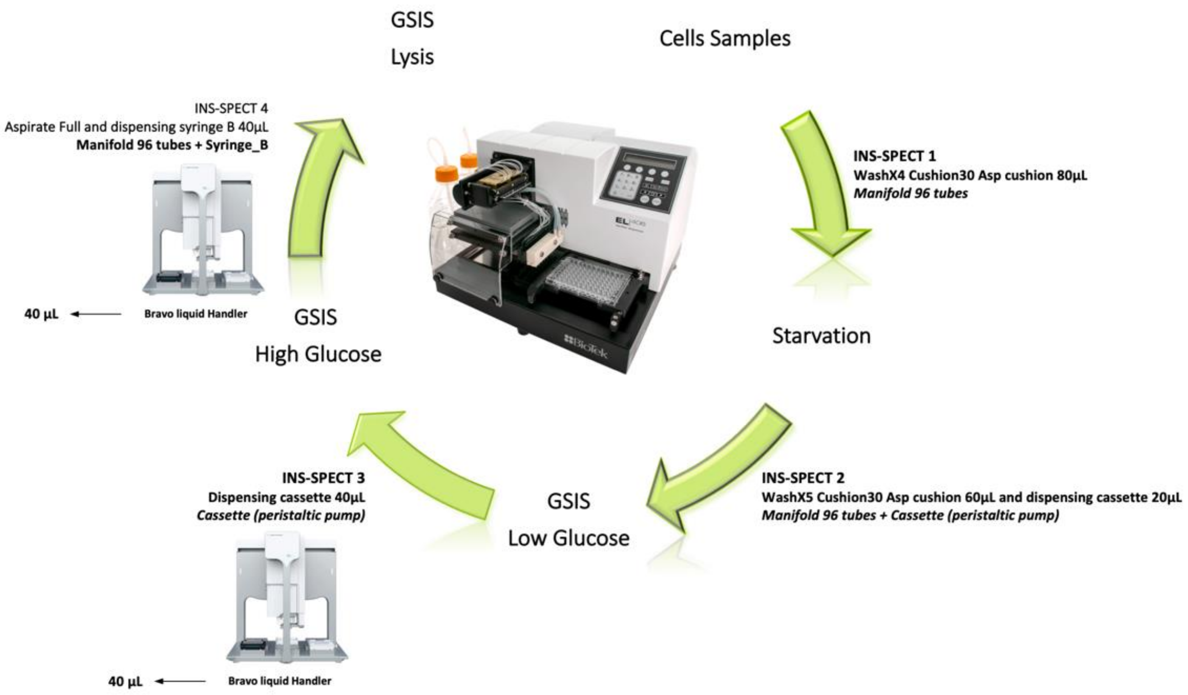

Figure S2: Schematic Representation of the GSIS pipeline.

Delannoy et al. Figure S3

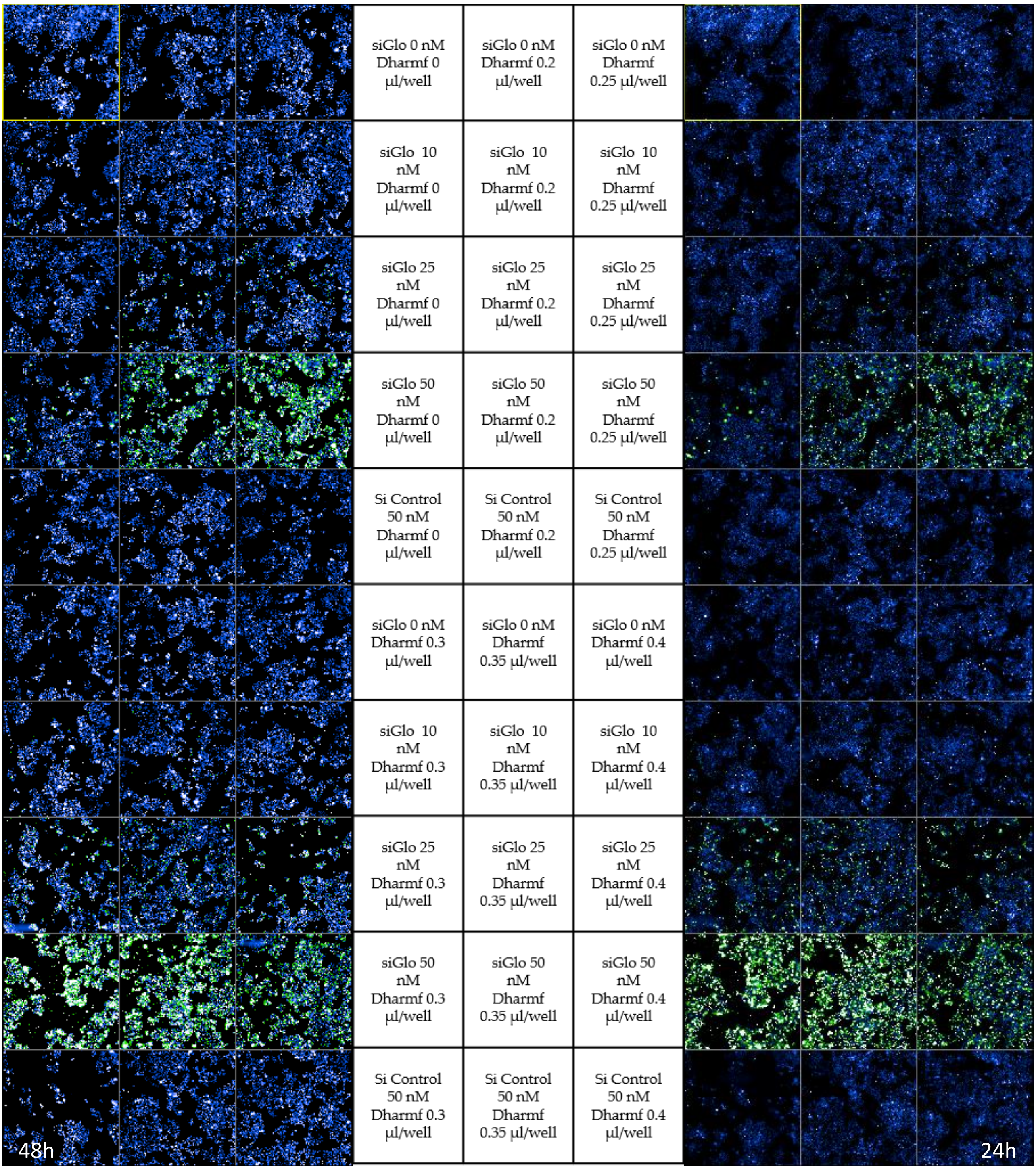

Figure S3: Efficiency tests for automated siRNA aproach. Confocal fluorescence microscopy images of cells transfected with siGLO and non-fluorescent siControl at different concentrations, time of incubation. Different concentrations for the transfectant reagents is also shown.

Delannoy et al. Figure S4

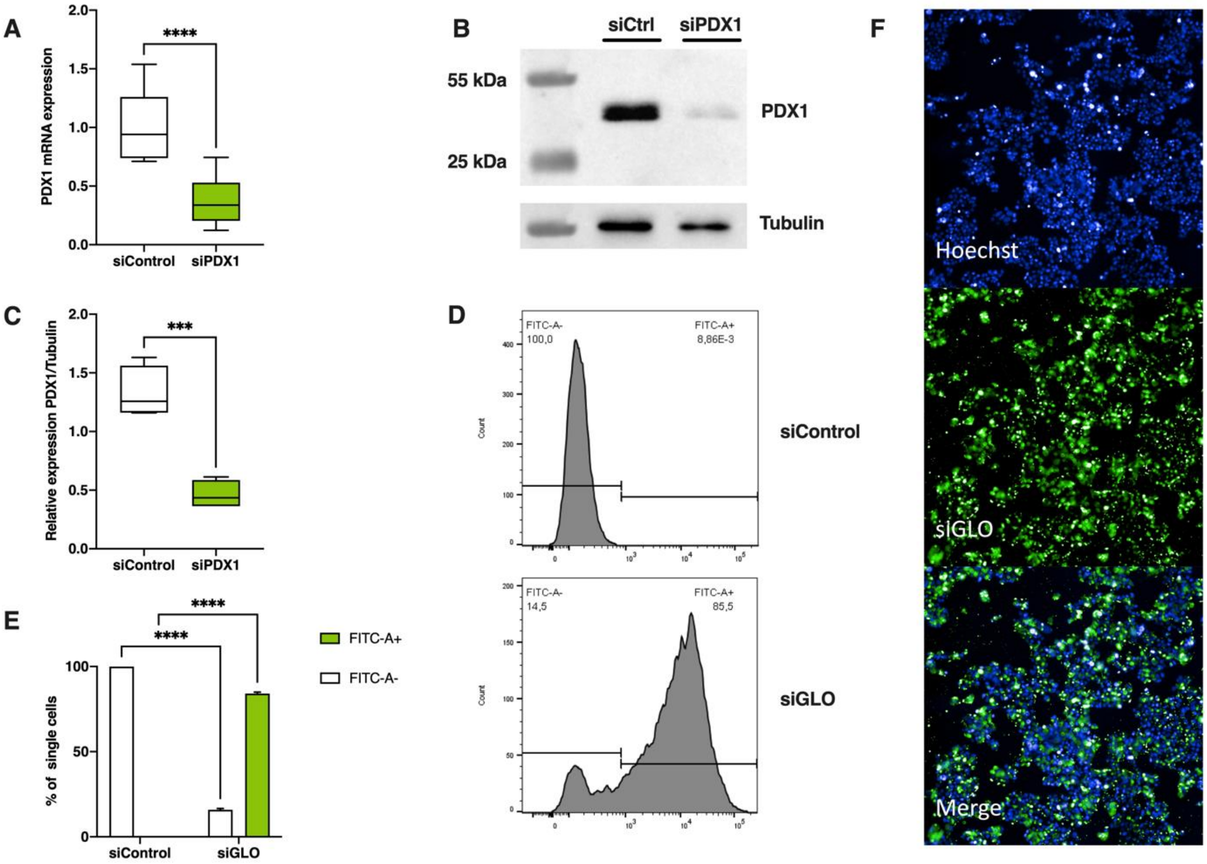

Figure S4: Efficiency of automated transfection of Min6 cells. (A) PDX1 mRNA expression from siCtrl and siPDX1 Min6 cells after automated reverse transfection; (B, C) Western blot showing mouse PDX1 protein level in Min6 cells after automated reverse transfection with siRNA control and siPDX1; (D) Histogram and (E) graphical representation of the number of FITC-A- and FITC-A + cells after transfection with siGLO. (F) Confocal fluorescence microscopy images of cells transfected with siGLO. Two-way ANOVA with Bonferroni posttest analyses. Error bars are ± SEM and n=3. *** p<0.001; **** p<0.0001.

Delannoy et al. Figure S5

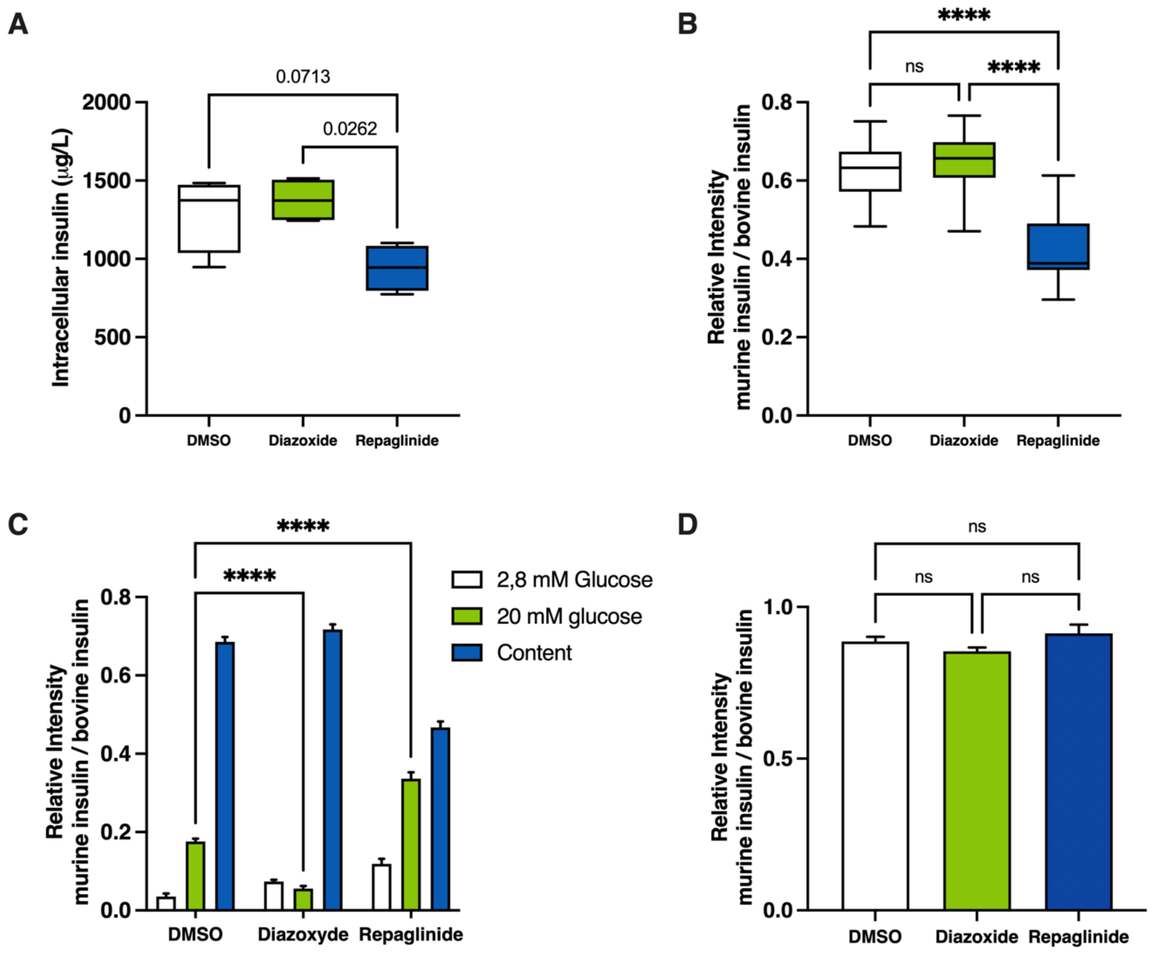

Figure S5: Insulin secretion analysis of Min6 cells treated with diazoxide and repaglinide by MALDI-TOF mass spectrometry. Quantification of insulin intracellular content of Min6 cells treated with DMSO, diazoxide 100µM and repaglinide 100nM by ELISA assay (A) and MALDI-TOF mass spectrometry (B, C). (D) Total insulin stored and secreted in min6 cells treated with DMSO, diazoxide 100µM and repaglinide 100nM measured through MALDI-TOF mass spectrometry. Two-way ANOVA with Bonferroni posttest analyses. Error bars are ± SEM and n=4 for ELISA, n=32 for MS. **** p<0.0001

Delannoy et al. Figure S6

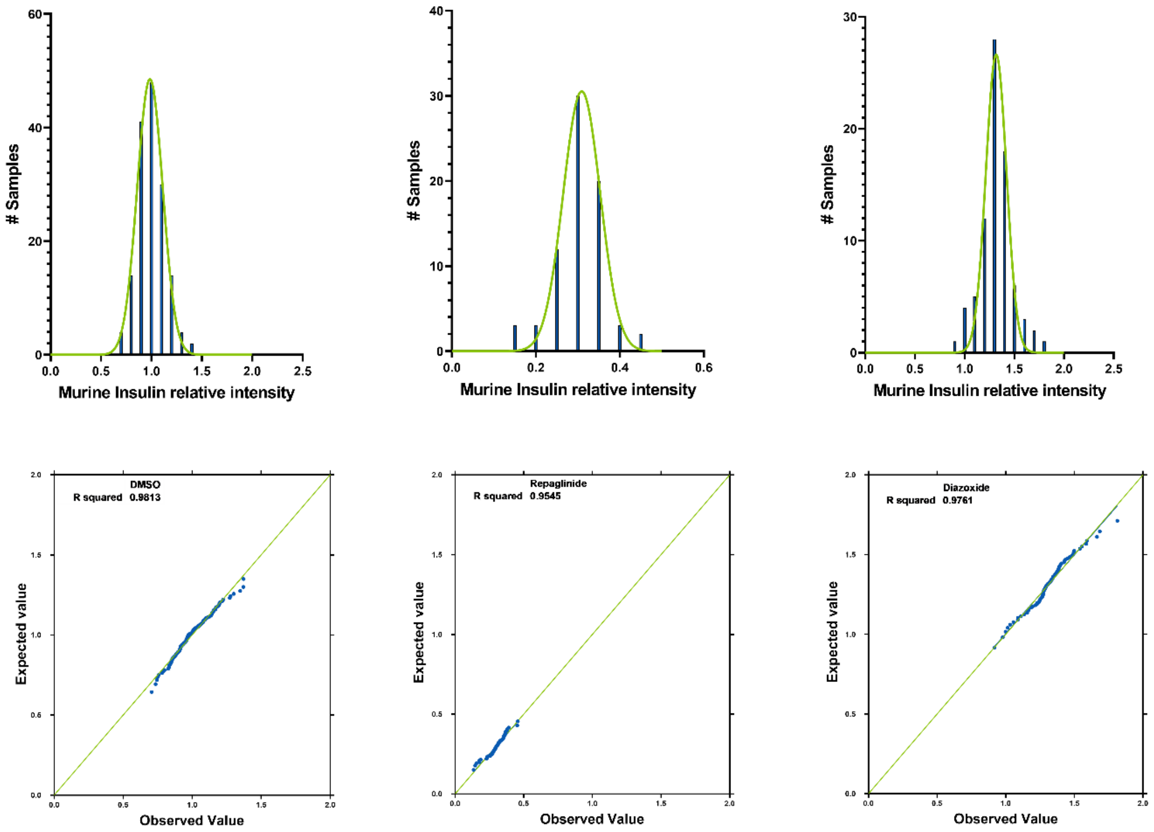

Figure S6: Normal distribution of analysis of intracellular insulin content from Min6 cells treated with DMSO, repaglinide and diazoxide. Histograms representing frequency distribution for murine insulin relative intensity (Upper panel). Simple linear regression of Normality and Lognormality Tests of Normality for murine insulin relative intensity (Lower panel). n=160 for DMSO and n=80 for Repaglinide and Diazoxide.

Delannoy et al. Figure S7

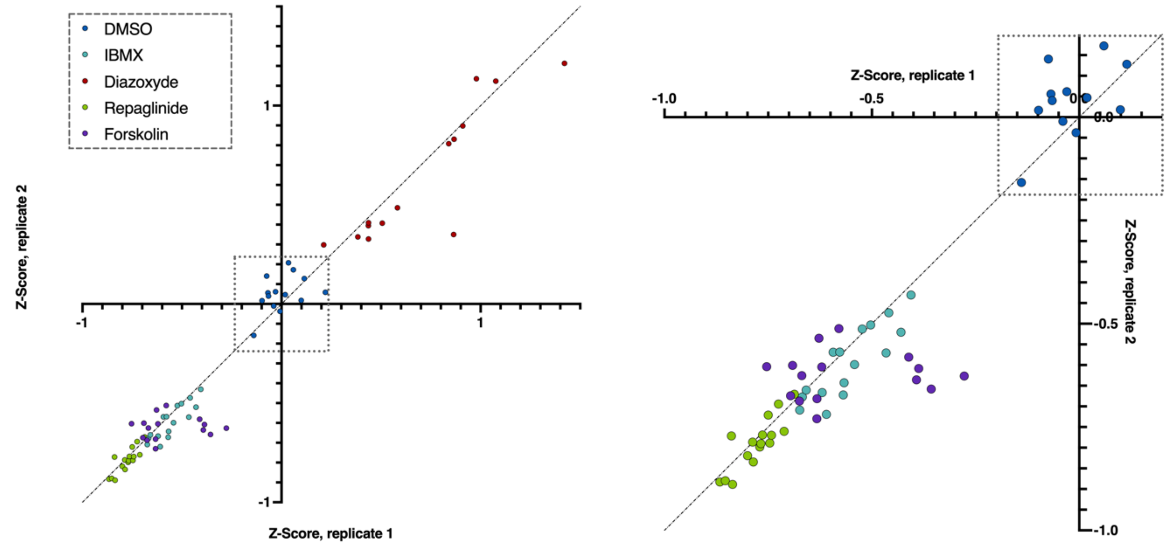

Figure S7: High-throughput analysis of intracellular insulin content of min6 cells treated with DMSO, IBMX, diazoxide, repaglinide and forskolin after glucose-stimulated insulin secretion.

Min6 cells were treated with three bioactives molecules known to induce insulin secretion, repaglinide (green), IBMX (light blue) and Forskolin (purple) whereas diazoxyde (red) was used as an inhibitor of insulin secretion
